# Diverse Effects of a Seven-Year Experimental Grassland Fragmentation on Major Invertebrate Groups

**DOI:** 10.1371/journal.pone.0149567

**Published:** 2016-02-18

**Authors:** Brigitte Braschler, Bruno Baur

**Affiliations:** Section of Conservation Biology, Department of Environmental Sciences, University of Basel, Basel, Switzerland; Weyerhaeuser NR, UNITED STATES

## Abstract

Habitat fragmentation is a major driver of biodiversity loss, but observed effects vary and may depend on the group examined. Time since fragmentation may explain some differences between taxonomical groups, as some species and thus species composition respond with a delay to changes in their environment. Impacts of drivers of global change may thus be underestimated in short-term studies. In our study we experimentally fragmented nutrient-poor dry calcareous grasslands and studied the response of species richness, individual density and species composition of various groups of invertebrates (gastropods, ants, ground beetles, rove beetles, orthoptera, spiders, woodlice) in 12 small (1.5 m * 1.5 m) and 12 large (4.5 m * 4.5 m) fragments and their corresponding control plots after 7 years. We further examined responses to fragmentation in relation to body size and habitat preferences. Responses to fragmentation varied between taxonomical groups. While spider species richness and individual density were lower in fragments, the opposite was true for an orthopteran species and woodlice. Species composition and β-diversity differed between fragments and control plots for some groups. However, the interaction treatment*plot size was rarely significant. Species with high occupancy rates in undisturbed control plots responded more negatively to the fragmentation, while species with large body size were relatively more abundant in fragments in some groups. No effect of the fragmentation was found for ants, which may have the longest lag times because of long-lived colonies. However, relationships between abundance and the species’ preferences for environmental factors affected by edge effects indicate that ant diversity too may be affected in the longer-term. Our results show the importance of considering different groups in conservation management in times of widespread fragmentation of landscapes. While species richness may respond slowly, changes in abundance related to habitat preferences or morphology may allow insights into likely long-term changes.

## Introduction

Biodiversity loss due to environmental change is a major concern. Habitat fragmentation is considered to be one of the most threatening drivers of environmental change for biodiversity [1, 2]. Habitat fragmentation has led to dramatic landscape changes during the previous century in many parts of the world [3]. Fragmentation leads to isolation of subpopulations and disturbs or alters interactions among species [4–10]. Small populations in small remnants have greater sensitivity to demographic stochasticity [11, 12] and typically experience reduced genetic variation [13]; effects that are further enhanced by isolation, which reduces recolonization after local extinctions [14, 15] and thus leads to altered species composition in fragments. Edge effects may further alter species composition through immigration of generalists from the matrix [16] and by providing a new habitat type with different microclimatic conditions [17–20].

Studies examining the effects of habitat fragmentation on biodiversity have included both surveys in fragmented landscapes with remnants of different size, age and degree of isolation (e.g. [21]) as well as experiments that artificially subdivided formerly continuous habitat [22]. In the early stages of an experiment, species composition in the newly created fragments represents a random subset of the local species pool, which then gradually shifts due to edge and other effects leading to local extinction. Therefore, fragmentation effects may become more apparent after some delay, with the lag time depending on the characteristics of the experimental design and the focal group of organisms. In long-lived species occupying larger fragments these lag times can be substantial. Thus, even older fragments may still have an extinction debt [21, 23, 24]. Studies using the ‘space for time’ substitution have also drawbacks including the lack of appropriate controls [25]. It follows that some fragmentation effects cannot be demonstrated in short-term experimental studies. It is therefore essential to compare short-term and long-term fragmentation effects in multiple groups within the same experimental setting.

We also need to relate the responses to species’ traits to improve our long-term predictions of the impacts of environmental change and identify groups likely affected by extinction debts. Even where substantial lag times exist, indications of future declines may be visible early on. Demographic changes like reduced recruitment [14], changes in predator-prey [4, 8], or herbivore-plant interactions [8, 26], or behavioral changes of pollinators leading to reduced genetic diversity and increased inbreeding [7, 27] may all be first steps along the way leading to declining species richness. Even highly mobile species may be affected when species with low mobility that show a response are involved in interactions with them. In long-lived species, a response in a shorter-lived species they interact with may be the first indication of future responses.

In this study we compare responses of seven groups of invertebrates to small-scale habitat fragmentation in the seventh year of a controlled experiment conducted in species-rich, nutrient-poor calcareous grasslands. The groups examined vary in trophic rank, size and mobility, and represent different branches from the tree of live. We firstly examine whether species density, individual density and species composition of the focal groups were differently affected by the experimental fragmentation. We expect that species or taxonomic groups with short generation length should be more influenced by grassland fragmentation than species or groups with longer generations, which may respond only with some delay. Furthermore, groups with species of high trophic ranks such as the predacious spiders may be more strongly affected because of their dependence on other groups than groups mainly consisting of species of low trophic ranks. Secondly, we examine whether body size or preferences for environmental conditions affected by edge effects can explain different responses between focal groups and among species within these taxonomic groups, and whether such relationships are also visible in groups whose diversity did not yet respond to the experimental fragmentation. Body size has previously been found to be important in fragmentation studies. Body size is related to the needs for habitat size, which makes it more likely that larger species show a negative response to fragmentation, especially in smaller fragments. Conversely, larger species tend also to be more mobile and may thus experience less isolation. This could lead to larger species being less strongly affected by fragmentation. In addition to isolation and habitat size, habitat quality may also be affected by fragmentation. Especially the edge zone of fragments is exposed to external influences altering microclimatic conditions and vegetation structure. Thus, species for which the conditions in the edge zone of fragments are suitable may show positive responses to the fragmentation and vice versa.

## Materials and Methods

### Study sites

**Fig 1:**
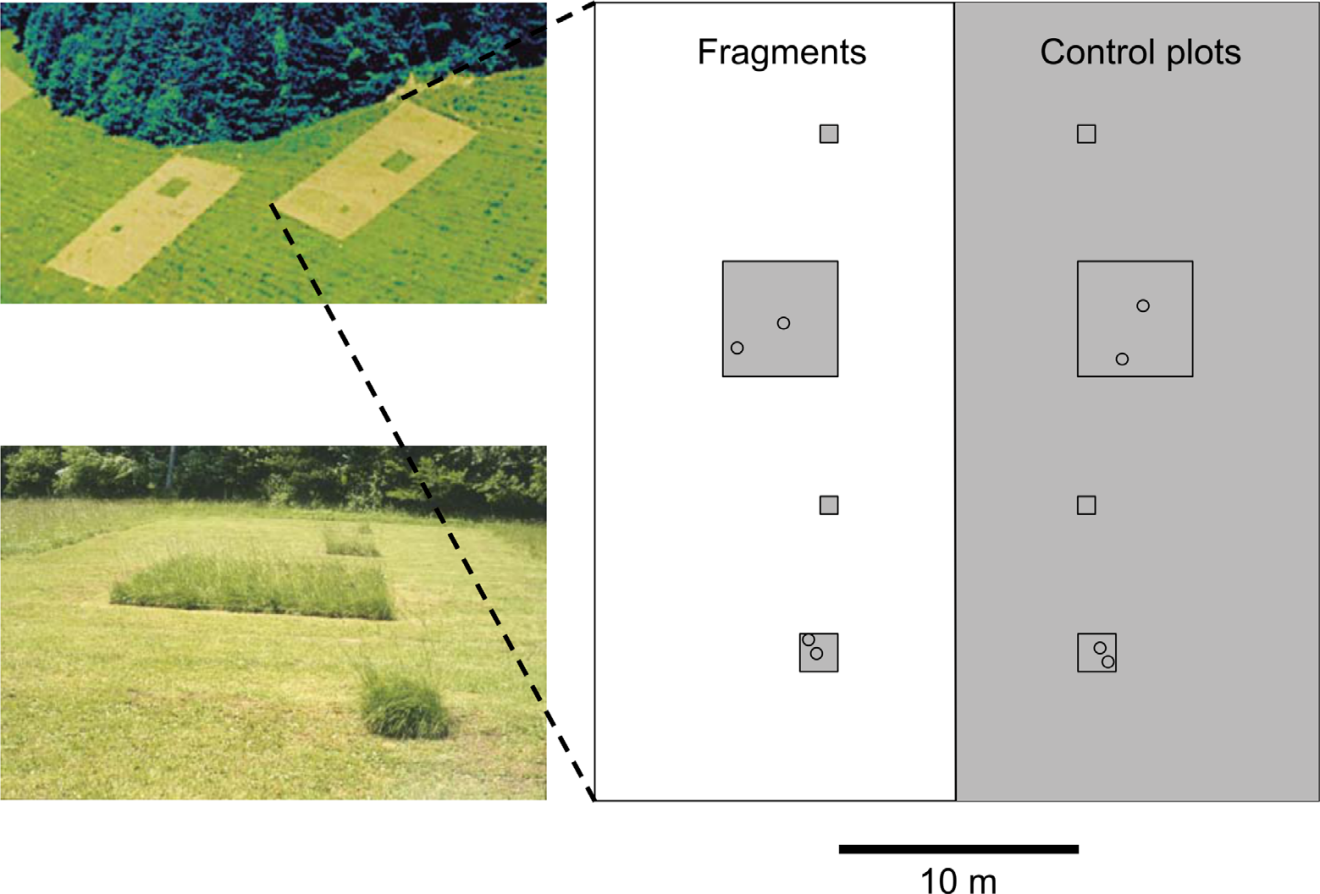
Experimental design. Twelve experimental blocks (29 m x 32 m) were distributed over three grasslands. Fragments were isolated by regularly mowing the surrounding matrix. Each block contained one large (4.5 m x 4.5 m), one small (1.5 m x 1.5 m) and two tiny (0.5 m x 0.5 m) fragments and corresponding control plots in unmown vegetation. The order of the fragment and control plot pairs within a block was randomized. The tiny plots were not considered in this study. Two pitfall traps (open circles) were set in each experimental plot, with one located near the edge and one in the core zone of the plot. Photo: M. Wurtz.

We chose a habitat type that has been heavily affected by habitat fragmentation and other drivers of environmental change like altered agricultural practices within Europe: the species-rich nutrient-poor semi-natural grasslands [3]. The three grasslands selected for the experiment were calcareous grasslands in the Swiss Jura Mountains near Movelier (Midpoint of site: 47.413558° N, 7.323867° E, 770 m a.s.l.; see also inset air photograph and photograph of fragments in Fig 1), Nenzlingen (47.448586° N, 7.567864° E, 510 m a.s.l.), and Vicques (47.363867° N, 7.426114° E, 590 m a.s.l.). The three sites were on slopes as is the case for most remaining nutrient-poor dry grasslands in the region. The site in Movelier was on a south-south-east-facing slope (inclination 20–22°), the one in Nenzlingen on a south-west-facing slope (19–22°) and the one in Vicques on a south-east-facing slope (15–27°). We rented the land for the fragmentation experiment and took over the management for 7 years in agreement with their owners. The three study sites were previously used as pasture for centuries, but were fenced throughout the experiment, and only mown at the end of the growing season in each year to prevent encroaching by woody plants. The study sites were parts of larger areas of Teucrio-Mesobrometum grasslands [28, 29], with grazing by cattle continuing in the parts that were not fenced of for our study. Soil depth was shallow in Nenzlingen and Vicques with the bedrock exposed in several places; contributing to the relative dryness of these grasslands. Bordering the sites was also mixed deciduous forest. The distances among sites ranged from 9.5 to 18.8 km. Mean annual temperatures for the sites ranges from 7.4 to 8.9°C and precipitation from 917 to 1104 mm (long-term climate data derived from the WorldClim database [30] which fit well with locally measured data in Nenzlingen). The sites are described in detail in Baur *et al*. [31].

### Fragmentation experiment

The experimental fragmentation was created in spring 1993. Grassland fragments were isolated by regularly mowing (7–11 times per year) the matrix around them throughout the growing season. Sward height of the matrix was held at 4–5 cm and cuttings from the mowing were removed. The mowing also prevented plants from flowering in the matrix and thus was removing some sources of food for invertebrates. A block design was implemented in which fragments were isolated by 5 m from the nearest unmown grassland, with corresponding control plots marked 5 m from the same edge within the continuous habitat (Fig 1). This distance was selected as representative of the width of the typical road in this region. The distances between the nearest edges of blocks within sites ranged from 25 to 135 m. The fragmented areas of the blocks contained fragments of three different sizes, separated by 5 m of mown matrix. However, the smallest fragments were not included in this study. The large fragments and control plots measured 4.5 x 4.5 m in size, while the smaller plots considered were 1.5 x 1.5 m (Fig 1). Three blocks were located in Movelier, five in Nenzlingen and four in Vicques. Further details on the experimental design can be found in Zschokke *et al*. [17].

### Sampling

Pitfall traps were set in 1999 during the seventh year of the fragmentation experiment. Two traps were placed in each plot. Trap location was randomized but one trap was close to the edge while the other was in the core zone of the experimental plot to standardize possible edge effects by ensuring that both individuals potentially affected by the fragment edge and those in more sheltered parts of the fragments were considered in each fragment (Fig 1). Edge effects themselves were not a focus of this study. The core zone was defined as starting 50 cm from the edge as previous work showed that two edge effects–increased temperature and plant biomass–did not exceed this distance [17, 18]. Additionally edge effects on ant nest dispersion were observed up to 40 cm inside the fragments [32]. Traps were 6.7 cm in diameter and partly filled with 10% glycerol solution, which prevents evaporation and is non-attractive to arthropods. Each trap was emptied of contents every two weeks throughout the period of highest invertebrate activity from 6 May to 12 August for a total of 7 collections. Contents were then sorted into broad taxonomic groups and identified to species by the authors and external experts. The following groups of invertebrates were chosen for analysis: gastropods (snails and slugs), ants, ground beetles, rove beetles, orthopterans, spiders and woodlice. Bycatch (myriapoda, some other beetles (mostly scarabidae), and a few small vertebrates (2 shrews and a few lizards)), which were not included in the analyses, comprised less than 0.5% of specimens. Very small arthropods like mites and springtails were not considered.

To avoid interfering with the population dynamics in the fragments, experiments conducted in this field experiment during the first 6 years used exclusively non-invasive methods (no animals were removed) and thus did not influence the species richness and abundance of invertebrates considered in this study.

### Body size

Body size is often considered as a factor in a species’ vulnerability to habitat fragmentation, though its effect may depend on spatial scale and other factors. Larger species need larger home ranges and more resources, and thus may more likely be absent from smaller fragments than smaller species. Conversely, however, larger species may also be more likely to cross the matrix and thus experiencing less isolation. We chose species-specific adult size (length of longest axis) as reported in the literature and web databases as a measure of body size. We transformed the data by calculating group means and then expressing the size of each species as a proportion of this group mean. In this way comparisons across groups became possible. In snails the mean shell size was considered, in slugs mean extended body length. Means were thus calculated for snails and slugs separately. For the other focal groups, maximum length was chosen as many sources reported a range for adult size rather than a mean, and the body size distribution for these species is unknown, rendering the use of a midpoint of the range arbitrary. In the case of woodlice only data on maximum size was available. Ants represent a special case as these social insects form colonies with different castes and some species are polymorphic within a caste as well. We decided to focus on the size of workers as males and queens spend most time within the nests and were rarely caught in our pitfall traps.

When the data on body size were used for analysis the standardized values are based on data from all species even when only frequent species are included in the respective analysis.

### Habitat preference

Edge effects alter the habitat quality in fragments, which in turn affects species composition through habitat preferences. Edges are exposed to wind and solar irradiation, which may affect plant growth and alter microclimatic conditions such as soil humidity and temperature in the edge zone of fragments. In our experiment, temperature and vegetation structure were altered along the edge [17, 18] as plant productivity and thus turf height and plant density were increased in fragments. Invertebrate species, which were well adapted to the original habitat type, thus were expected to be negatively affected by fragmentation-related habitat changes. We chose occupancy in control plots as an indication of suitability of the original habitat. This measure is assumed to provide a baseline for habitat suitability of the continuous grassland in absence of detailed niche information. However, other factors may determine rarity in some species and thus contribute to the variation in such a relationship.

For some focal groups more detailed information on habitat preferences was available. For ants the indicator value system developed by Seifert [33], including preferences for temperature, humidity, and vegetation density, was used. For gastropods information on humidity preferences was available [34]. Similarly information on humidity preferences was available for rove beetles from H. Luka.

Due to the close proximity of forest to the examined grasslands (Fig 1) some typical forest species were caught. All analyses were repeated excluding all strict forest species (forest species defined based on habitat preferences in the literature). However, this did not change any findings and therefore only analyses based on the full data set are presented.

### Statistical analyses

For some focal groups (ants, gastropods), we compared our data with species lists, which were obtained using different collection methods in the same study sites over several years. All ant species and all but one (rare) gastropod species previously recorded in the experimental plots using non-destructive sampling methods were also found in pitfall traps. The exception among the gastropods is a rock-dwelling species, of which one individual has been translocated from close by rocky outcrops. For both ants and gastropods some, mostly rare, species were only caught in pitfall traps. These species were represented by few vagrant individuals usually living in nearby situated forests. Furthermore, two cryptic ant species, which are mostly active in soil crevices as specialist hunters of small soil arthropods, are easily missed in nest or bait surveys. In fact, one of these species is so rarely recorded that this constituted the first record of workers of this species for Switzerland [35]. These findings confirmed that most species known to be present in the study sites were caught in the pitfall trap survey. We therefore decided to base our analyses on the observed data rather than a derived measure of species richness accounting for not detected species.

Data from both traps of a plot were averaged for analyses using a two-step approach. First, we averaged data on the number of species and the number of individuals for the two traps per plot for each collection event. However, a few pitfall trap samples failed (15 out of 672). In these cases we used the data from the only functioning trap instead of a two-trap average. In a second step, data from all seven collection periods were then averaged per plot. Thus, we obtained species density (number of species per trap and collection period) and individual density (number of individuals per trap and collection period) for each plot. These densities per sample were used for the models examining the effects of fragmentation treatment, plot size, and their interaction on the numbers of species and individuals. The models considered the nested structure of the experiment including the random factors study site and block in addition to the above listed fixed factors. The dependent variables were log_10_(x+1)-transformed when needed. In contrast to the species density used in the analyses, the term “total species richness” is used to refer to the combined species list based on all samples collected in a plot or treatment. It is not used in analyses but presented for reference and as an indication of the size of the species pool.

For analyses, which examined the response to fragmentation of individual density of different species separately, we considered only frequent species, defined as those present in at least 10 out of 48 experimental plots (fragments or control plots of either size combined). All other species were considered to be rare and their response to fragmentation was analyzed together using paired-tests with species as replicates and log_10_(x+1)-transformed data on total abundances in fragments respectively control plots.

Species composition may be affected by the experimental fragmentation even in cases when species density and individual density are not. To visualize such changes non-metric multidimensional scaling (nMDS) was used. In this ordination approach an as close as possible representation is made of the pairwise dissimilarity in species composition among experimental plots. The method is based on rank order data giving information about which is the most similar and next most similar plot to a specific plot rather than information on the magnitude of the differences. For visualization, each plot is placed in a multidimensional space according to the rank order distances between plots. The procedure is iterative. The arrangement of plots is then projected progressively on multidimensional spaces with fewer dimensions till reaching a two-dimensional representation pictured here. Thus, plots whose symbols are shown in close proximity are considered to have a more similar species composition than those whose symbols are farther apart. nMDS does not directly provide a statistical test of whether species composition of certain groups of sites are different. For this reason we used permutational multivariate analysis of variance (PERMANOVA) to test whether species composition differed between fragments and control plots. PERMANOVA too is a non-parametric method. The individual density data were used to calculate Bray-Curtis dissimilarity, which has good properties for ecological studies as it is not affected by additions or removals of species that are not present in two plots or by the addition of other plots. This distance matrix was used as basis for nMDS plots using function *metaMDS* and for PERMANOVA using function *adonis* in the package vegan [36] for R [37]). The latter analyses were done with the treatments nested within the factor study site as nMDS plots showed clusters of experimental plots depending on the study site. The *strata* option was thus used to account for the differences in species composition among sites by ensuring that randomizations were only made within each site. Woodlice were not present in some of the experimental plots and these plots were thus removed from nMDS or Permanova analyses focusing on this group. While ground beetles were present in all experimental plots, there was one plot with only one specimen, which furthermore represented a species unique to the study. Analyses on ground beetles were thus repeated without this outlier and the resulting nMDS plot was used because of the extreme distance between the outlier and the other plots.

Fragmentation may lead to either more homogeneous species composition if the species in the fragments are a nested subset of the species pool in the continuous grassland, or it may enhance differences provided the species turnover is higher in fragments than in control plots. To examine species turnover we used *adonis* to study β-diversity (β_z_ from Koleff et al. [38] using the package vegan [36] for R [37]) in fragments and control plots and analyzed it using the function *betadiver* in package vegan, which provides indices of pairwise beta diversity including β_z_, followed by the permutational multivariate analysis of variance *adonis* in package *vegan*.

A species’ response to fragmentation will depend on its environmental preferences. For ants, gastropods and rove beetles we had information on humidity preferences, and for ants we had also information on preferences for plant density and temperature. For these groups we used models relating responses by species to the fragmentation in relation to known indicator values for habitat preferences. As a species’ response to fragmentation we used the t-value for the fragmentation effect from the full models described above relating the individual density of species to the fragmentation treatment, plot size and their interaction. Only frequent species were considered.

A similar approach was followed to relate a species response to fragmentation to their body size; a trait that was assumed to be related to dispersal ability and the size of habitat required.

As species’ responses may be similar due to common ancestry, we corrected the models relating the response to fragmentation to body size or habitat preferences for relatedness by using taxonomic levels as a proxy for phylogenetic distances. An actual phylogeny covering all species found in the present study is not available, however, taxonomic levels should be correlated. We therefore included taxonomic information as nested random factors in the models. For analyses focusing on separate focal groups, genus and family or subfamily were used. Subfamily replaced the level family in the insect groups ants, ground beetles and rove beetles, which each consisted of only one family. For analyses using data from all focal groups combined, we added group as the highest level and used family or subfamily depending on the group each species belonged to alongside genus. Generally, including taxonomic information into the models had little influence on the findings as our reduced dataset, which only included the frequent species, had few genera that were represented by more than one or two species. A negative value for the response to fragmentation (t-values, respective residuals for the t-values from the separate models) indicates a negative response of a particular species to the experimental fragmentation, while a positive t-value indicates that a species reached a higher individual density in fragments than control plots.

Analyses were done in R [37] (packages vegan [36], nlme [39]).

## Results

### Species richness and density of focal groups

In total 230 species belonging to the 7 focal groups were sampled: 188 species (81.7%) were found in fragments and 197 (85.7%) in control plots with 155 species (67.4%) shared by both. Total species richness, species density and individual density varied between focal groups (Table 1). Ants accounted for nearly half of specimens collected (49%), while having moderate species richness. Orthoptera were represented by only one very abundant species (*Gryllus campestris* Linnaeus 1758), while spiders represented 45% of all species including many rare species.

**Table 1:** Species richness, species density and individual density of focal groups^a^^,^^b^ in fragments (F) and control plots (C).

Focal group	Total species richness^c^		Species density^d^		Individual density^e^	
	F	C	F	C	F	C
Gastropoda (snails and slugs)	21	18	2.26 ± 0.70	1.99 ± 0.64	5.54 ± 2.55	4.58 ± 2.16
Hymenoptera						
Formicidae (ants)	24	22	2.86 ± 0.76	2.94 ± 0.68	13.68 ± 5.06	15.52 ± 5.16
Coleoptera						
Carabidae (ground beetles)	25	29	0.38 ± 0.24	0.35 ± 0.22	0.46 ± 0.34	0.40 ± 0.26
Staphilinidae (rove beetles)	27	31	0.40 ± 0.29	0.49 ± 0.29	0.56 ± 0.54	0.73 ± 0.76
Orthoptera (grasshoppers and crickets)	1	1	0.27 ± 0.22	0.11 ± 0.15	0.96 ± 1.20	0.31 ± 0.62
Araneae (spiders)	91	88	3.00 ± 1.18	4.03 ± 1.40	5.59 ± 3.06	9.43 ± 4.50
Isopoda (woodlice)	4	5	0.40 ± 0.29	0.22 ± 0.21	1.04 ± 1.08	0.38 ± 0.44

^a^ Four insect families, the crustacean order isopoda, spiders, as well as gastropods (snails and slugs) were examined. Bycatch not represented by these focal groups formed less than 0.5% of the samples.

^b^ Response variables represent averages of the two traps per plot averaged over the seven 2-week collections throughout the season, except for total species richness which is the combined total of all species observed in any fragment or control plot. Mean values ± 1 SD are shown. N = 24 for species density and individual density.

^c^ Total number of species observed in fragments or control plots

^d^ Number of species per trap and sampling period

^e^ Number of individuals per trap and sampling period

Overall neither species density nor individual density differed between fragments and control plots (Tables 1 and 2). However, the experimental fragmentation affected some focal groups and several species within the focal groups. Spider species density and individual density was lower in fragments than in control plots, while woodlouse species density and individual density was higher in fragments (Table 2). Similarly, the individual density of *Gryllus campestris* was higher in fragments. In contrast, species density and individual density of gastropods, ants, ground beetles, and rove beetles did not differ between fragments and control plots (Table 2). However, for gastropods a significant treatment*plot size interaction was observed, as the small fragments had higher gastropod species density than either the large fragments or the control plots (Tables 1 and 2). For none of the other groups were significant plot size or treatment*plot size interactions observed (Table 2). Repeated analyses with the plot size and interaction terms removed from the model did not change the findings for the treatment effect.

**Table 2:** Summaries from the full models^a^ for species density and individual density of all groups combined and each focal group separately showing results for the fixed effects fragmentation treatment (fragments vs. control plots), plot size (large vs. small) and the interaction treatment*plot size^b^.

	Treatment			Plot size			Interaction		
	df	t	p	df	t	p	df	t	p
Species density									
All Groups	11	-1.65	0.13	22	-0.27	0.79	22	0.64	0.53
Ants	11	-0.86	0.41	22	-0.71	0.48	22	0.16	0.87
Orthopterans	11	2.17	0.0528	22	-0.10	0.92	22	1.64	0.12
Ground beetles	11	0.93	0.37	22	0.94	0.36	22	-0.72	0.48
Rove beetles	11	-1.06	0.31	22	0.10	0.92	22	-0.22	0.83
Gastropods	11	-0.47	0.65	22	-1.51	0.15	22	**3.10**	**0.0052**
Spiders	11	**-3.37**	**0.0063**	22	0.76	0.45	22	-0.63	0.54
Woodlice	11	**2.59**	**0.0250**	22	0.92	0.37	22	-0.99	0.34
Individual density									
All Groups	11	-0.88	0.40	22	0.56	0.58	22	-0.77	0.45
Ants	11	-1.13	0.28	22	0.72	0.48	22	-0.01	0.99
Orthopterans	11	**2.78**	**0.0180**	22	0.71	0.48	22	-0.80	0.43
Ground beetles	11	1.23	0.24	22	0.91	0.37	22	-0.96	0.35
Rove beetles	11	-1.26	0.23	22	-0.60	0.56	22	0.57	0.58
Gastropods	11	0.77	0.46	22	-1.68	0.11	22	1.00	0.33
Spiders	11	**-3.89**	**0.0025**	22	1.25	0.22	22	-1.81	0.08
Woodlice	11	**2.78**	**0.0180**	22	0.71	0.48	22	-0.80	0.43

^a^ The full model also accounted for the nested structure of the design and included the random factors site and block. Dependent variables were log_10_-transformed or log_10_(x + 1)-transformed for analysis except for ant species density.

^b^ Significant effects are shown in bold font. Positive t-values for treatment indicate groups that reached higher densities in fragments than in control plots, while negative t-values indicate groups whose densities were lower in fragments. The significant interaction term for gastropod species density was caused by the smaller fragments having increased species density than the larger fragments, while no difference was found between control plots of different size.

Pitfall traps remove individuals, which may result in biased estimates of the fragmentation effect after continuous sampling over a longer period. However, seasonal differences in activity would be neglected if sampling is conducted only during a short period. We explored whether our data shows seasonal differences by repeating analyses for the early period (first three collecting events combined) and late period (last three collecting events combined) (S2 Table). Findings for the early period were generally similar to those for the full dataset. However, several groups showed different responses for the late period. Although overall species density and individual density was lower in the late period, this was not true for all groups and several species were only present during the late period. Indeed ground beetles showed a positive response to fragmentation late in the season that was not observed early in the season.

Some of the 79 frequent species showed responses that deviated from the overall pattern of their focal group (S1 Table). The overall individual density of spiders was lower in fragments than in control plots. However, only 4 out of 37 frequent spider species showed this response when analyzed separately. There were two spider species with higher individual density in fragments than control plots, while the remaining 28 common spider species did not respond to fragmentation. Similarly, while there was no overall effect on gastropod or ground beetle individual density, a few species in both of these groups differed significantly in individual density between fragments and control plots. One out of 4 frequent ground beetle species, *Carabus violaceus purpurascens* Fabricius 1787, benefited from fragmentation. Interestingly, *C*. *violaceus purpurascens* was the second largest ground beetle species observed in the study sites. Similarly, the large invasive slug *Arion vulgaris* Moquin-Tandon 1855 benefited from fragmentation. In contrast, the tiny snail *Punctum pygmaeum* (Draparnaud 1801), with a mean shell diameter of only 1.4 mm, was negatively affected by fragmentation. For the remaining 10 frequent gastropod species the fragmentation effect was not significant. In line with their whole focal groups, the individual density of none of the 15 frequent ant or 7 frequent rove beetle species responded to the fragmentation, while the only frequent woodlouse species, *Trachelipus rathkii* (Brandt 1833), benefited from it. Again *T*. *rathkii* is a relatively large species. Overall only for 11 (14%) of 79 frequent species, 6 of them being spiders, a significant treatment effect was observed. However, for a few species significant plot size effects or significant treatment*plot size interactions were observed, indicating that for some species the fragmentation effect was scale-dependent.

The total abundance of rare species was greater in control plots than in fragments (paired t-test on log_10_(x+1)-transformed data with species as replicates: t_149_ = 2.34, P = 0.0193). Relative distribution in fragments and control plots, however, varied widely among rare species. In the case of gastropods, 7 out of 10 rare species had nearly two thirds or more individuals in fragments (> 64% of individuals in fragments), while the remaining 3 species were more abundant in control plots. In contrast to the preference for fragments found in gastropods, 3 out of 4 rare woodlouse species had more individuals in control plots, with only one species being more abundant in fragments. The rare species of the other focal groups showed similar abundances in fragments and control plots (percentage of rare species with more than 50% of individuals in control plots respective fragments: ants: 55.6% vs 33.3%, ground beetles: 46.7% vs 36.7% rove beetles: 54.8% vs 35.5%, spiders: 54.5% vs 36.4%; percentages do not add up to 100% because several of the rare species were exactly evenly distributed and counted towards the total when calculating percentages). T-Tests revealed no significant differences when considering the focal groups separately.

### Relationship between response to fragmentation and body size

The frequent gastropod, ground beetle and woodlouse species, which responded positively to the fragmentation, were among the largest species in their respective groups, though the same was not true for the two frequent spider species, which also were more abundant in fragments. To examine whether body size can be used as a predictor of a species’ response to fragmentation, we used the t-values for the treatment effect derived from the GLMM models for the individual density of separate species as a measure of the strength and direction of the species’ response to the experimental fragmentation. Overall we found a significant relationship between a species’ size and its response to the experimental fragmentation, with the larger species responding more positively and the smaller species more negatively to the fragmentation (t_24_ = 2.25, P = 0.034). Considering the taxonomical groups separately, the relationship was significant for gastropods (t_1_ = 25.15, P = 0.025). No relationship between response to experimental fragmentation and body size was found for ants, rove beetles or spiders. For ground beetles, orthopterans and woodlice too few species were recorded for separate analysis. However, both the only orthopteran species and the only woodlouse species abundant enough for separate analysis were relatively large, as well as significantly more abundant in fragments than in control plots, thus supporting the overall pattern.

### Relationship between response to fragmentation and habitat preferences

**Fig 2:**
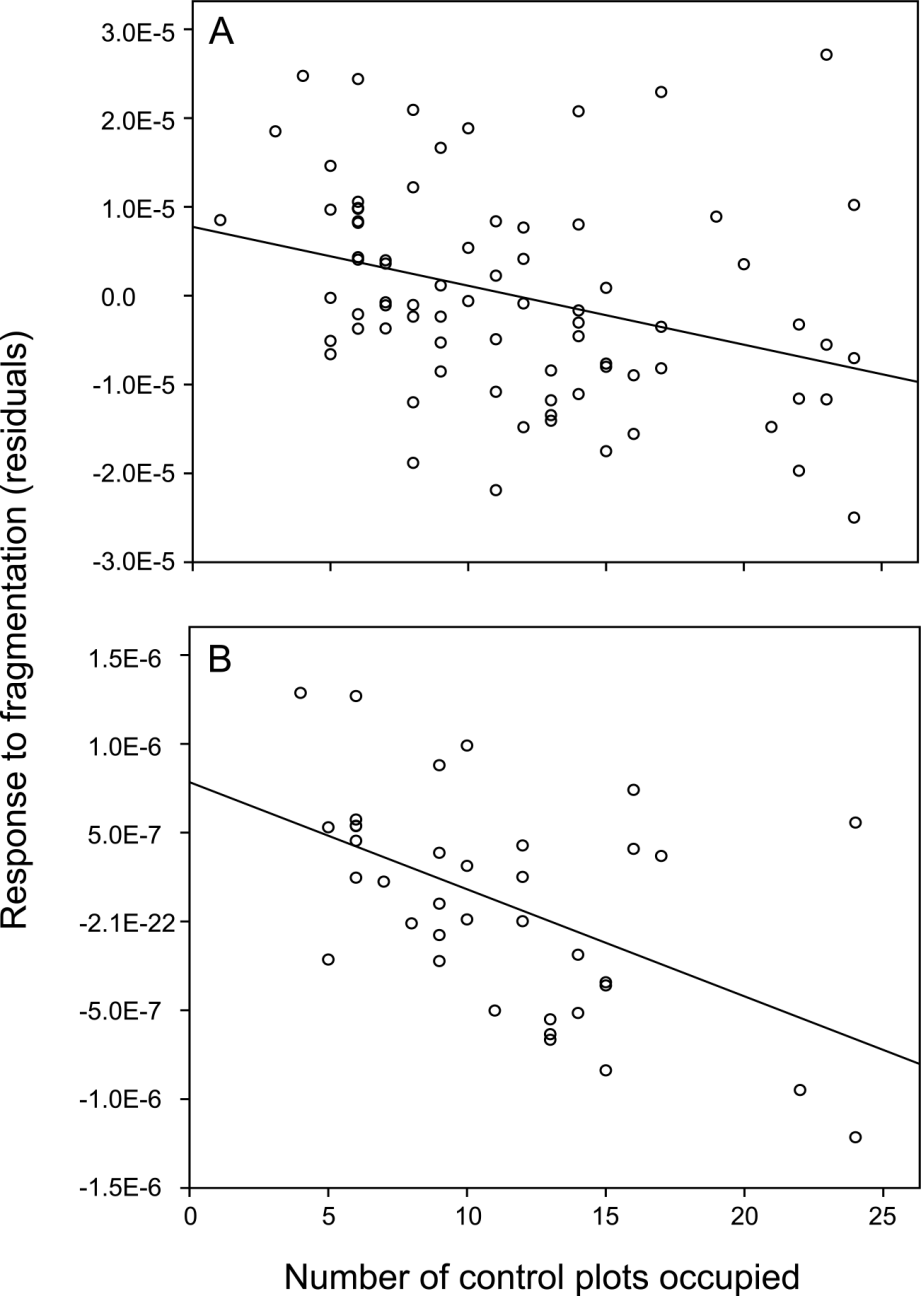
**Relationship between response to the experimental fragmentation and occupancy rates in control plots for (A) all frequent species and for (B) frequent spider species.** Response to the fragmentation was measured as the t-value for the treatment effect from separate full models on the individual density of the species (see statistical analyses). A negative residual for the t-value indicates a negative response of a particular species to the experimental fragmentation, while a positive residual for the t-value indicates a species that reached a higher individual density in fragments than control plots. Significance tests from models including taxonomic information (see main text for details): Frequent species of all focal groups: t_24_ = -3.19, P = 0.004; frequent spider species: t_13_ = -4.74, P = 0.0004.

Species for which the conditions in the original habitat were highly suitable are expected to show high occupancy rates in control plots. Such species, however, may be more vulnerable to fragmentation-related changes in the environment. We therefore examined whether these species are differently affected by habitat changes resulting from the experimental fragmentation than species, which showed lower occupancy in control plots. Considering all groups together, there was a negative relationship between occupancy in control plots and the species’ response to fragmentation (Fig 2). This was largely a result of the relationship for spiders (Fig 2), as no significant relationship was found for the other focal groups.

**Fig 3:**
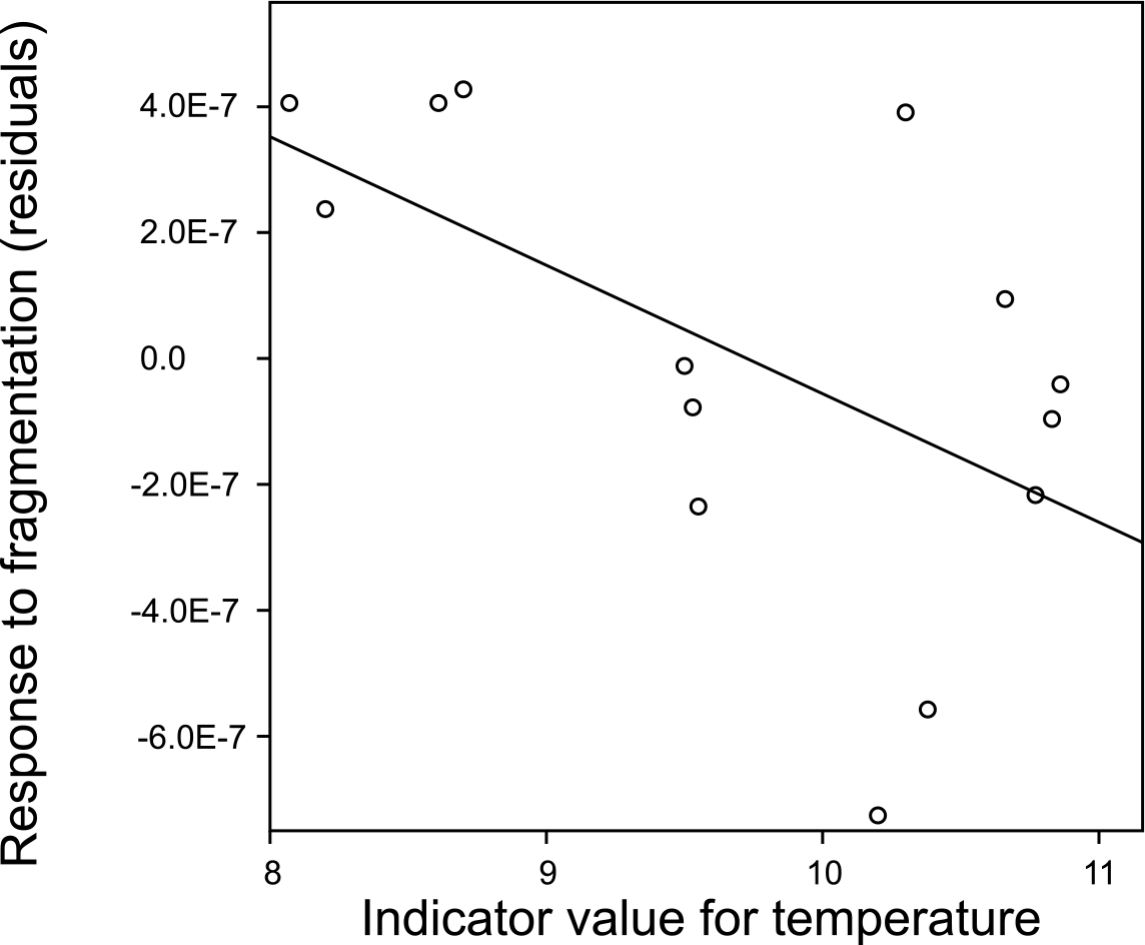
Response of frequent ant species to the experimental fragmentation depending on their preferences for temperature. The response to fragmentation is expressed by the t-value for the effect of the fragmentation treatment on the individual density of the separate species in full models (see statistical analyses). Indicator values following Seifert [33] were used to define a species’ temperature preferences. Indicator values relate ant distributions within Germany to the indicator values for the plants in the sites of occurrence following Ellenberg [52]. A negative residual for the t-value indicates a negative response of a particular species to the experimental fragmentation, while a positive residual for the t-value indicates a species that reached a higher individual density in fragments than control plots. Significance test from model including taxonomic information (see main text for details): t_7_ = -2.67, P = 0.032.

For ants detailed indicator values for their habitat preferences are available. While no significant relationships between a particular ant species’ response to the experimental fragmentation and their indicator values for humidity or plant density were found, ant species which preferred lower temperatures were relatively more abundant in the fragments (Fig 3). This is in line with increased plant productivity measured in the edge zone of fragments as the denser and higher plants may provide more shade [18].

No significant relationships between the responses to fragmentation of frequent gastropod or rove beetle species and their preferences for humidity were found (Spearman correlations with the t-values for the fragmentation effect from the GLMMs and the ordered humidity preferences). Only four ground beetle species were available for separate analysis of the fragmentation effect. The only ground beetle species with a significant response (positive) to the fragmentation was mesophilous (no clear preference for humidity conditions), while the species with the most negative–though not significant–response to the fragmentation was xerophilous (preferring dry conditions).

### Species composition

**Fig 4:**
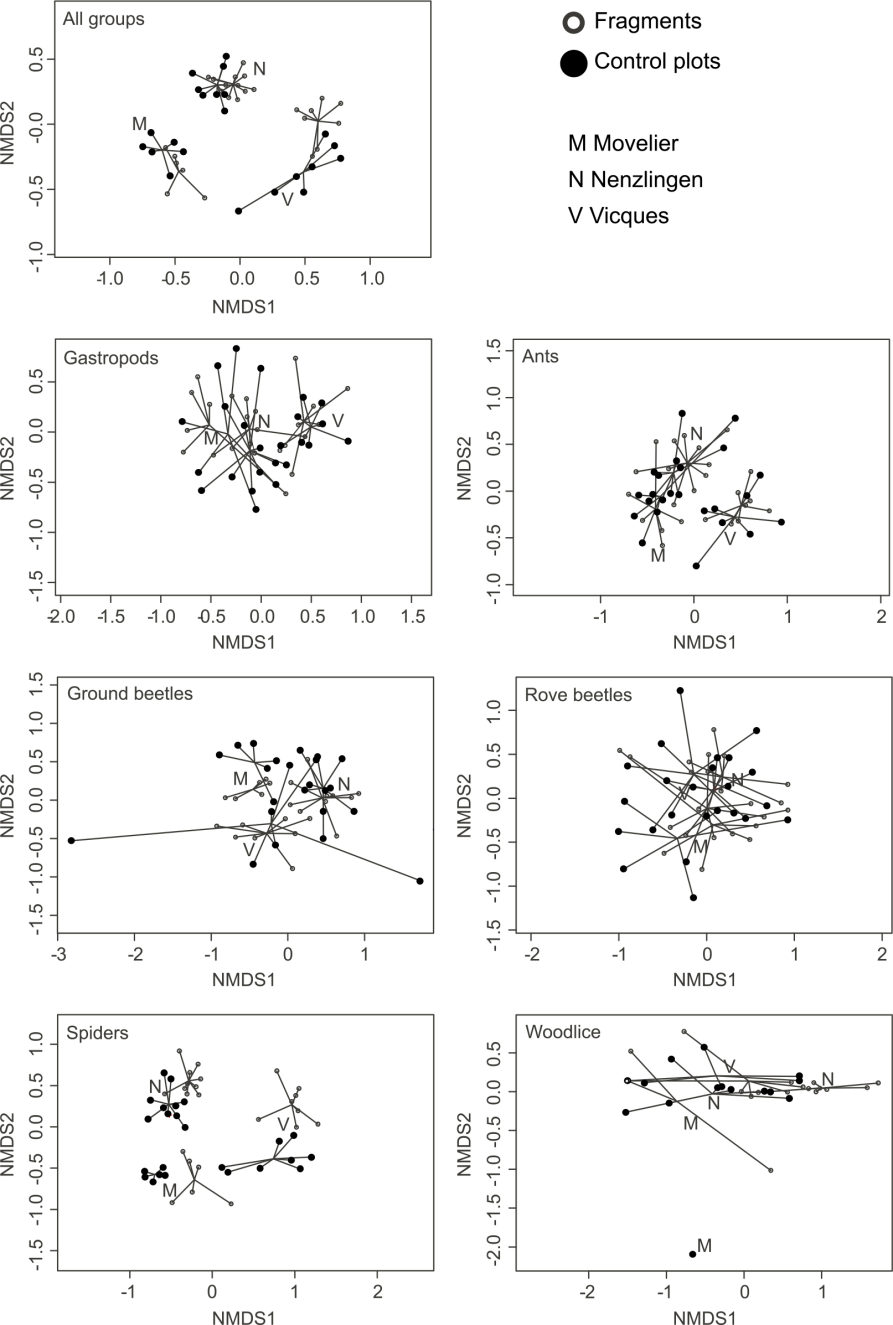
nMDS plots for overall species composition and for species composition of the focal groups separately. Orthopterans are included in the overall analysis but not shown separately as the group consisted of only one species. One extreme outlier (a control plot in Vicques) was removed for the ground beetle nMDS plot as this experimental plot only had one specimen, which was the only representative of its species. Woodlice were absent in several plots and these plots were removed from analysis for this group.

Non-metric multidimensional scaling plots (nMDS plots) showed that species composition varied between the three study sites for most focal groups examined (Fig 4). Ninty-eight of the 230 species (43%) occurred in only one of the sites, and a further 67 (29%) in only two of the three sites. For this reason we nested the fixed factors within site for analyses of species composition using PERMANOVA (using *strata* in function *adonis* in R package vegan [36]).

While some degree of separation in species composition between fragments and control plots of the same site could be seen in nMDS plots for some focal groups, the distributions of fragments and control plots in the nMDS plots did strongly overlap for other focal groups (Fig 4). The fragmentation treatment effect on species composition was significant for overall species composition (PERMANOVA, R^2^ = 0.04, F_1,44_ = 2.05, P = 0.0498). Differences between fragments and control plots were stronger when considering spiders (R^2^ = 0.07, F_1,44_ = 3.33, P = 0.0149) and ground beetles (R^2^ = 0.05, F_1,44_ = 2.39, P = 0.0299; with outlier removed: R^2^ = 0.05, F_1,43_ = 2.39, P = 0.0149) separately, and to a lesser extent for woodlice (R^2^ = 0.10, F_1,33_ = 3.67, P = 0.0448). However, the species composition of gastropods, ants and rove beetles did not vary significantly between fragments and control plots. Plot size and the interaction term were never significant.

Species composition in fragments may differ from that of control plots in continuous habitat because some species disappeared from the fragments or some species benefited from the new conditions. In a situation where species disappear it may always be the same species, which are not able to cope with the changed conditions. In this situation, the species composition of fragments represents a nested subset of the species found in control plots. However, if new species are added to fragments, which otherwise are not present in the continuous habitat, or when fragments have reduced richness but the identity of those species which manage to persist varies among fragments, then we expect a higher species turnover among different fragments than among different control plots. In the first case, we could thus expect β-diversity of fragments to be reduced, while in the second case it may be higher than in control plots. Indeed β-diversity (β_z_ from Koleff et al. [38]), the factor treatment was significant for gastropods (R^2^ = 0.04, F_1,44_ = 1.75, P = 0.0448) and ground beetles (R^2^ = 0.04, F_1,44_ = 2.18, P = 0.0050; with the outlier removed: R^2^ = 0.05, F_1,43_ = 2.18, P = 0.0100). In all these cases β-diversity was increased in fragments when compared to control plots. No significant difference to the species turnover in control plots was found for ants, rove beetles, spiders or woodlice. When overall species composition was analyzed the fragmentation did not have an effect on β-diversity (R^2^ = 0.02, F_1,44_ = 1.15, P = 0.21). Again the factor plot size and the interaction term were not significant in the analyses.

## Discussion

A survey of the existing literature on the effects of habitat fragmentation shows a wide variety of biodiversity outcomes [22]. Where multiple groups have been examined in the same setting this finding was often supported with different focal groups reacting differently to the fragmentation (e.g. [10, 17]). Our experiment examined different invertebrate groups covering several distinct invertebrate lineages as well as different trophic levels. While the diversity of most of the groups responded to the experimental fragmentation, it did so in different ways. Spider species richness decreased in line with expectations based on the assumed detrimental effect of habitat fragmentation on biodiversity. However, other focal groups showed no such response or even the opposite response. Similarly for some groups, species composition differed between fragments and control plots while for others this was not the case. Traits could explain some of the responses, but their importance likewise differed between groups, with body size, preferences for microclimatic conditions, and frequency in control plots all related to a species’ relative density in fragments for some groups but not for others.

Habitat fragmentation can affect species through isolation, reduced patch size, or changed habitat quality, including edge effects. The importance of these factors will vary among groups depending on their physiology, morphology and behavior. Some species will have perceived the surrounding matrix in our experiment as a strong barrier, while for others the matrix may indeed not have been a barrier at all but rather a preferred habitat, which could fortify populations in the fragments through edge effects. For example, the matrix contained nests of some ant species and a high burrow density of the cricket *G*. *campestris*. However, while the orthopteran was indeed reaching higher individual density in fragments when compared to control plots, no such effect was seen in those ant species that also had nests in the matrix. In contrast, rather than showing preference for the xerothermic conditions seen in the short-turfed matrix, those ant species with a relative more positive response to the experimental fragmentation were those which preferred relatively lower temperatures and thus likely rather benefited from the dense vegetation in the edge zone of fragments, which provided shade, than the conditions prevailing in the surrounding short-turfed matrix.

Even species that could not use the matrix itself as habitat will not necessarily have been detrimentally affected by it provided their mobility is high, as was the case for some of the species or focal groups examined. The study focused on soil surface-active species. However, some of these species are also capable of flight and thus of long-distance dispersal. In particular, rove beetles are able to fly, which facilitates crossing the matrix. Indeed, the diversity of rove beetles was not affected by the fragmentation.

Dispersal by air is also an option for other groups, which are not commonly regarded as flyers. Young queens of many ant species have wings and can thus found new colonies far from their original colony [33]. However, new colonies may also be founded through budding from older colonies in some species; a method resulting in much shorter dispersal distances [33]. Another mobile group are juvenile spiders dispersing by ballooning. Nonetheless, spider species density in fragments was clearly reduced. Most carabids are reluctant or unable to fly. However, exceptions exist, e.g. the tiger beetle *Cicindela campestris* Linnaeus 1758, which was present in our experiment. Carabid species density and individual density did not differ between fragments and control plots, although changes in species composition were observed. For this group changes in habitat characteristics may have been more important than the isolation, even though most species would have dispersed solely by walking. Gastropods are incapable of flight themselves, but smaller species may be transported by the wind while attached to leaf litter. Thus, even snail species typical for the nearby forests may reach fragments in this way. Nonetheless, earlier work in the same experiment studying population dynamics of 6 snail species indicated that the matrix represented a barrier [15]. That study showed that local extinctions were more frequent in fragments and that the four small species considered had altered re-colonization frequencies in smaller fragments [15].

For many of the studied species, including the sole orthopteran, the woodlice, larger gastropods, and many carabids, active dispersal is limited based on how far they can disperse on foot. Other common long-distance dispersal modes such as attachment to grazing mammals were not possible in our experiment as the study sites were fenced to prevent access to large animals.

The observed relationship between body size and response to fragmentation indicated that isolation was an important factor in determining a species’ response to the experimental fragmentation. Large-bodied species were relatively more frequent in fragments when compared to corresponding control plots than was the case for smaller species of their group. This may indicate that the barrier effect of the matrix was reduced for larger species, which likely are better dispersers. Other factors may also affect whether species can cross the matrix. Previous observations showed that individuals of a few gastropod species were active under mild conditions during the winter and thus would be able to cross the matrix during favorable winter days [15, 40]. This comparatively reduced barrier to dispersal may have contributed to the increased species density of gastropods in small fragments. As most invertebrates are not active during winter, however, most other species examined were not able to benefit from this opportunity.

In contrast to the distance to the continuous habitat, which was held constant in our experiment, the effects of different patch sizes were an integral part of our experimental design. We expected treatment * plot size interactions, as for some species the smaller fragments would be too small for supporting them. Furthermore, edge effects are relatively more important in smaller fragments as they have a larger proportion of edge habitat. Indeed, earlier work showed that fragments had an increased plant biomass in the edge zone as well as different plant species composition, resulting in altered vegetation structure and turf height [18]. Such changes in the vegetation then further affected light conditions and temperature in the fragments [17, 18] and may have affected humidity. Thus, the experimental fragmentation may change habitat suitability for many invertebrate species, with the effects expected to be more pronounced in small fragments.

Contrary to our expectations, we found little evidence of a scale-dependence of the fragmentation effect even though edge effects seem to have contributed to the observed fragmentation effects. A significant treatment*plot size interaction was observed for gastropod species density and for the individual density of a few species. However, considering species density, individual density or species composition, in almost any analyses the interaction term treatment * plot size was not significant. This indicates that for most species the two fragment sizes–albeit differing in area by factor 9—did present similarly suitable or unsuitable habitat. Isolation, which was equal for both, and the presence of edge habitat rather than its proportion, may have been more important in determining a species’ presence or density in fragments than the area of the fragment.

For most focal groups examined either species density, individual density or species composition were affected by the experimental fragmentation. Ants and rove beetles were the exception. Ant colonies are long-lived entities compared to most solitary arthropods. This may mean that lag effects are especially pronounced in this group. However, the group might have still responded to the fragmentation had the experiment been run over an even longer period. This is indicated by the relationship between the response to the fragmentation and the ant species’ relative preferences for temperature. Furthermore, earlier work in the same experiment using different methods revealed that the behavior of ants in fragments did change (aphid tending [8], nest dispersion and persistence [32], and numerical dominance at baits [9] all differed between fragments and control plots). For all these reasons, effects on ant species composition can be expected over the longer-term. Our survey was done in the seventh year of the experiment (with an earlier survey based on nest counts in the fourth year already showing no differences in density for the examined ant species [17]).

Habitats become subdivided at multiple spatial scales. Fragmentation ranges from small breaks in otherwise homogeneous habitat to widely distributed fragments in a hostile matrix. Consequently, fragmentation experiments have been conducted at different spatial scales depending on the habitat requirements and activity ranges of various taxonomic groups and species examined. Large spatial scales are considered when studying forest fragmentation and mobile groups such as mammals and birds [41–43]. In contrast, experiments focused on openland invertebrates or small vertebrates have often focused on smaller spatial scales [4, 22]. The smallest spatial scales were used in an experiment examining the fragmentation of moss patches inhabited by tiny arthropods [44]. In our mesochosm field experiment we focused on a relatively small scale, isolating fragments of a size more commonly found in urban gardens than in rural areas. We focused on relative immobile groups of soil-surface active invertebrates rather than the often more mobile pollinators and herbivorous insects of the plant canopy. Many species examined were also small enough to have sizeable populations in our plots. The smallest species of several groups had a maximum adult size of 2 mm or were even smaller (ants 2.2 mm, gastropods 1.6 mm, ground beetles 7.0 mm, rove beetles 2.2 mm, spiders 1.5 mm). The absence of significant fragmentation treatment * plot size interactions indicates that even the smaller fragments may have been large enough for most of the species examined.

The width of the matrix (5 m) was chosen to represent the width of a local road, as they are frequent in the agricultural landscape of this region. This allows a comparison with findings of other studies conducted on either side of roads in real landscapes. Our matrix may represent a relatively mild barrier to most species when compared to that of paved roads or other sealed surface or novel ecosystems. Interestingly, however, previous results from our fragmentation experiment confirm findings on isolation effects of roads. For example, the matrix with no flowering plants around the fragments influenced the foraging behavior of bumblebees [7], which resulted in an increased frequency of self-fertilization and changes in genetic diversity in plants growing in fragments [27]. Similar alterations in foraging behavior of bumblebees were recorded in verges of roads [45]. Furthermore, the matrix in our experiment functioned as partial barrier for certain snail species [15] as found in the verges of real roads [46, 47] Moreover, most invertebrate groups examined showed responses to the experimental fragmentation. We also found effects of fragmentation on the dynamics and genetic diversity of plants [6, 48] and on the behavior, species density or individual density in animal groups that were not treated in this paper because they are not typically collected by pitfall traps (e.g. aphids [8], bumblebees [7], butterflies [17], and other orthopterans [17, 49]). This indicates that the spatial scale of our fragmentation experiment is of relevance for various plant and invertebrate species but not for all [50].

## Conclusions

Spiders were the most strongly and most negatively affected focal group. Given the high species density in the examined grasslands and important ecological functions of this group this is of concern. Some other groups showed different responses including benefiting from the fragmentation as in the case of gastropods, woodlice, and the *G*. *campestris*, or no response as in the case of ants, whose species composition may be slow to respond due to long-lived colonies. Body size, frequency in the control plots, and preferences for climatic conditions all could help explain response to fragmentation in some focal groups but not in others. The strong negative effect on spider species density and individual density would thus not necessarily have been predicted based on monitoring programs focusing on some of the other groups examined. This highlights the dangers of biodiversity studies focusing on just a small part of the overall diversity, unless the validity of the observed taxa as indicator taxa for overall biodiversity has been tested [51]. Focal groups in biodiversity monitoring in practical conservation are often selected at least partially based on available expertise. Based on our results this approach may have its limitations and care should be taken to establish the correlation of the response by the chosen indicator groups with the response of other important parts of the local biodiversity. Additionally, by going beyond simple surveys of species density and including traits or interactions among species, we may be able to predict longer-term changes in focal groups whose diversity lags behind the environmental changes.

## Supporting Information

S1 File Raw data. (XLS)

S1 Table Summaries for separate species analyses. (DOCX)

S2 Table Seasonal differences in fragmentation effects. (DOC)

S1 Text Methods and sources for habitat preferences of species. (DOCX)

S2 Text Species list. (DOCX)

## References

[1] Saunders DA, Hobbs RJ, Margules CR. Biological consequences of ecosystem fragmentation: review. Conserv Biol. 1991; 5: 18–32.

[2] Collinge SK. Effects of grasslands fragmentation on insect species loss, colonization, and movement. Ecology. 2000; 81: 2211–2226.

[3] WallisDeVries MF, Poschlod P, Willems JH. Challenges for the conservation of calcareous grasslands in northwestern Europe: integrating the requirements of flora and fauna. Biol Conserv. 2002; 104: 265–273.

[4] Kareiva P. Habitat fragmentation and the stability of predator-prey interactions. Nature. 1987; 326: 388–390.

[5] Kruess A, Tscharntke T. Habitat fragmentation, species loss, and biological control. Science. 1994; 264: 1581–1584. 17769603 10.1126/science.264.5165.1581

[6] Groppe K, Steinger T, Schmid B, Baur B, Boller T. Effects of habitat fragmentation on choke disease (*Epichloë bromicola*) in the grass *Bromus erectus*. J Ecol. 2001; 89: 247–255.

[7] Goverde M, Schweizer K, Baur B, Erhardt A. Small-scale fragmentation affects pollinator behaviour: experimental evidence from the bumblebee *Bombus veteranus* on calcareous grasslands. Biol Conserv. 2002; 104: 293–299.

[8] Braschler B, Lampel G, Baur B. Effects of experimental small-scale grassland fragmentation on the population dynamics of aphids. Oikos. 2003; 100: 581–591.

[9] Braschler B, Baur B. Experimental small-scale grassland fragmentation alters competitive interactions among ant species. Oecologia. 2005; 143: 291–300. 15619095 10.1007/s00442-004-1778-x

[10] Rossetti MR, Gonzalez E, Salvo A, Valladares G. Not all in the same boat: trends and mechanisms in herbivory responses to forest fragmentation differ among insect guilds. Arth-Plant Int. 2014; 8: 593–603.

[11] Harrison S. Local extinction in metapopulation context: an empirical evaluation. Biol J Linn Soc. 1991; 42: 73–88.

[12] Holsinger KE. Demography and extinction in small populations In: Young AG, Clarke GM, editors: Genetics, Demography and Viability of Fragmented Populations. Cambridge: Cambridge University Press; 2000 pp. 55–74.

[13] Krauss J, Schmitt T, Seitz A, Steffan-Dewenter I, Tscharntke T. Effects of habitat fragmentation on the genetic structure of the monophagous butterfly *Polyommatus coridon* along its northern range margin. Mol Ecol. 2004; 13: 311–320. 14717889 10.1046/j.1365-294x.2003.02072.x

[14] Hanski I. Patch-occupancy dynamics in fragmented landscapes. Trends Ecol Evol. 1994; 9: 131–135. 10.1016/0169-5347(94)90177-5 21236796

[15] Stoll P, Oggier P, Baur B. Population dynamics of six land snail species in experimentally fragmented grassland. J Anim Ecol. 2009; 78: 236–246. 10.1111/j.1365-2656.2008.01478.x 19120604

[16] Stamps JA, Buecher M, Krishnan VV. The effects of edge permeability and habitat geometry on emigration from patches of habitat. Am Nat. 1987; 129: 533–552.

[17] Zschokke S, Dolt C, Rusterholz H-P, Oggier P, Braschler B, Thommen GH, et al Short-term responses of plants and invertebrates to experimental small-scale grassland fragmentation. Oecologia. 2000; 125: 559–572. 28547226 10.1007/s004420000483

[18] Dolt C, Goverde M, Baur B. Effects of experimental small-scale habitat fragmentation on above-ground and below-ground plant biomass in calcareous grasslands. Acta Oecol. 2005; 27: 49–56.

[19] Aguilar R, Ashworth L, Galetto L, Aizen MA. Plant reproductive susceptibility to habitat fragmentation: review and synthesis through meta-analysis. Ecol Lett. 2006; 9: 968–980. 16913941 10.1111/j.1461-0248.2006.00927.x

[20] Stangler ES, Hanson PE, Steffan-Dewenter I. Interactive effects of habitat fragmentation and microclimate on trap-nesting Hymenoptera and their trophic interactions in small secondary rainforest remnants. Biodiv Conserv. 2015; 24: 563–577.

[21] Bommarco R, Lindborg R, Marini L, Öckinger E. Extinction debt for plants and flower-visiting insects in landscapes with contrasting land use history. Divers Distr. 2014; 20: 591–599.

[22] Debinski DM, Holt RD. A survey and overview of habitat fragmentation experiments. Conserv Biol. 2000; 14: 342–355.

[23] Kuussaari M, Bommarco R, Heikkinen RK, Helm A, Krauss J, Lindborg R, et al Extinction debt: a challenge for biodiversity conservation. Trends Ecol Evol. 2009; 24: 564–571. 10.1016/j.tree.2009.04.011 19665254

[24] Essl F, Dullinger S, Rabitsch W, Hulme PE, Pyšek P, Wilson JRU, et al Delayed biodiversity change: no time to waste. Trends Ecol Evol. 2015; 30: 375–378. 10.1016/j.tree.2015.05.002 26028440

[25] Pickett STA. Space-for-time substitution as an alternative to long-term studies In: Likens GE, editor. Long-term Studies in Ecology. New York: Springer; 1989 pp 110–135.

[26] Stoll P, Dolt C, Goverde M, Baur B. Experimental habitat fragmentation and invertebrate grazing in a herbaceous grassland species. Basic Appl Ecol. 2006; 7: 307–319.

[27] Rusterholz H-P, Baur B. Delayed response in a plant-pollinator system to experimental grassland fragmentation. Oecologia. 2010; 163: 141–152. 10.1007/s00442-010-1567-7 20155288

[28] Zoller H. Studien an *Bromus erectus*-Trockenrasengesellschaften in der Nordwestschweiz, speziell im Blauengebiet. Ber Geobot Inst ETH. 1947; 1946: 51–81.

[29] Schläpfer M, Zoller H, Körner C. Influences of mowing and grazing on plant species composition in calcareous grassland. Bot Helv. 1998; 108: 57–67.

[30] Hijmans RJ, Cameron SE, Parra JL, Jones PG, Jarvis A. Very high resolution interpolated climate surfaces for global land areas. Int J Climat. 2005; 25: 1965–1978.

[31] Baur B, Joshi J, Schmid B, Hänggi A, Borcard D, Stary J, et al Variation in species richness of plants and diverse groups of invertebrates in three calcareous grasslands of the Swiss Jura mountains. Rev Suisse Zool. 1996; 103: 801–833.

[32] Braschler B, Baur B. Effects of experimental small-scale grassland fragmentation on spatial distribution, density and persistence of ant nests. Ecol Entomol. 2003; 28: 651–658.

[33] Seifert B. Die Ameisen Mittel- und Nordeuropas. Tauer: Lutra–Verlags- und Vertriebsgesellschaft; 2007.

[34] Falkner G, Obrdlik P, Castella E, Speight M. Shelled Gastropoda of Western Europe. Munich: Friedrich-Held_Gesellschaft; 2001.

[35] Braschler B. Neue Aspekte zur Verbreitung von *Pyramica baudueri* (Emery, 1875) (Hymenoptera, Formicidae). Mitt Entomol Ges Basel. 2002; 52: 139–142.

[36] Oksanen J, Blanchet FG, Kindt R, Legendre P, Minchin PR, O’Hara RB, et al. vegan: Community Ecology Package. R package version 2.0–10. 2013. Available: http://CRAN.R-project.org/package=vegan.

[37] R Core Team. A language and environment for statistical computing. Vienna: Austria; 2012. Available: http://www.R-project.org/

[38] Koleff P, Gaston KJ, Lennon JJ. Measuring beta diversity for presence-absence data. J Anim Ecol. 2003; 72: 367–382.

[39] Pinheiro J, Bates D, DebRoy S, Sarkar D, R Core Team. _nlme: Linear and Nonlinear Mixed Effects Models_. R package version 3.1–117. 2014. Available: http://CRAN.R-project.org/package=nlme.

[40] Oggier P. Circadian and year-round activity of the land snails *Candidula unifasciata* and *Helicella itala* in grasslands of the Swiss Jura mountains. Malakol Abh. 1998; 19: 89–101.

[41] Andrén H. Effects of habitat fragmentation on birds and mammals in landscapes with different proportions of suitable habitat: a review. Oikos. 1994; 71: 355–366.

[42] Wiens JA. Habitat fragmentation: island v landscape perspectives on bird conservation. Ibis. 1994; 137 (Suppl): 97–104.

[43] Radford JQ, Bennett AF, Cheers GJ. Landscape-level thresholds of habitat cover for woodland-dependent birds. J Biol Conserv. 2005; 124: 317–337.

[44] Gonzalez A, Chaneton EJ. Heterotroph species extinction, abundance and biomass dynamics in an experimentally fragmented microecosystem. J Anim Ecol. 2002; 71: 594–602.

[45] Bhattacharya M, Primack RB, Gerwein J. Are roads and railroads barriers to bumblebee movement in a temperate suburban conservation area? Biol Conserv. 2003; 109: 37–45.

[46] Baur A, Baur B. Are roads barriers to dispersal in the land snail *Arianta arbustorum*? Can J Zool. 1990; 68: 613–617.

[47] Wirth T, Oggier P, Baur B. Effect of road width on dispersal and genetic population structure in the land snail *Helicella itala*. Z Ökol Naturs. 1999; 8:23–29.

[48] Joshi J, Stoll P, Rusterholz H-P, Schmid B, Dolt C, Baur B. Small-scale experimental habitat fragmentation reduces colonization rates in species-rich grasslands. Oecologia. 2006; 148: 144–152. 16429312 10.1007/s00442-005-0341-8

[49] Braschler B, Marini L, Thommen GH, Baur B. Effects of small-scale grassland fragmentation and frequent mowing on population density and species diversity of orthopterans: a long-term study. Ecol Entomol. 2009; 34: 321–329.

[50] Baur B, Erhardt A. Habitat fragmentation and habitat alteration: principle threats to many animal and plant species. Gaia. 1995; 4: 221–226.

[51] Niemelä J, Baur B. Threatened species in a vanishing habitat: plants and invertebrates in calcareous grasslands in the Swiss Jura mountains. Biodivers Conserv. 1998; 7: 1407–1416.

[52] Ellenberg H. Vegetation Mitteleuropas mit den Alpen in ökologischer, dynamischer und historischer Sicht, 5. Aufl. Stuttgart: Ulmer; 1996.

