# Supplementary material for: Diverse Effects of a Seven-Year Experimental Grassland Fragmentation on Major Invertebrate Groups
Source: PLoS One. 2016 Feb 18;11(2):e0149567. doi: 10.1371/journal.pone.0149567 (PMC4758731; doi:10.1371/journal.pone.0149567)
Supplement: S1 Table — (DOCX) [file pone.0149567.s002.docx]

**Supporting information for Braschler & Baur “Diverse Effects of a Seven-Year Experimental Grassland Fragmentation on Major Invertebrate Groups”**

**S1 Table. Summaries for separate species analyses.** Summaries of the full models for the effects of the fixed factors fragmentation treatment, plot size, and the interaction treatment*size on individual densities for species separately. The full models also accounted for the nested structure of the design and included the random factors site and block. Models were only run for the 79 frequent species, which occurred in at least 10 plots. Individual densities were calculated as number of individuals per trap and collection period. Only results for factors with p < 0.1 are shown. Data was log_10_(x+1)-transformed for analysis.

|  | **Treatment** | | | **Size** | | | **Interaction** | | |
| --- | --- | --- | --- | --- | --- | --- | --- | --- | --- |
|  | df | t | p | df | t | p | df | t | p |
| **Gastropods** |  |  |  |  |  |  |  |  |  |
| *Arion vulgaris* Moquin-Tandon 1855 | 11 | **2.88** | **0.0150** |  |  |  | 22 | -2.01 | 0.0567 |
| *Deroceras reticulatum* (O.F. Muller 1774) |  |  |  | 22 | **2.24** | **0.0355** |  |  |  |
| *Helix pomatia* Linnaeus 1758 | 11 | 1.90 | 0.0833 |  |  |  |  |  |  |
| *Nesovitrea hammonis* (Ström 1765) |  |  |  |  |  |  | 22 | 1.81 | 0.0842 |
| *Punctum pygmaeum* (Draparnaud 1801) | 11 | **-2.69** | **0.0209** | 22 | **-2.59** | **0.0166** | 22 | 2.02 | 0.0559 |
| *Trochulus sericeus* (Draparnaud 1801) |  |  |  |  |  |  | 22 | 1.88 | 0.0739 |
| *Vertigo pygmaea* (Draparnaud 1801) |  |  |  | 22 | **-2.57** | **0.0174** | 22 | 2.01 | 0.0567 |
|  |  |  |  |  |  |  |  |  |  |
| **Ants** |  |  |  |  |  |  |  |  |  |
| *Formica rufibarbis* Fabricius 1793 |  |  |  | 22 | **-2.11** | **0.0465** |  |  |  |
| *Tapinoma erraticum* (Latreille 1798) |  |  |  |  |  |  | 22 | -1.89 | 0.0722 |
| *Tapinoma subboreale* Seifert 2012 | 11 | -2.10 | 0.0593 |  |  |  |  |  |  |
|  |  |  |  |  |  |  |  |  |  |
| **Ground beetles** |  |  |  |  |  |  |  |  |  |
| *Calathus fuscipes* (Goeze 1777) |  |  |  |  |  |  | 22 | 1.98 | 0.0601 |
| *Carabus monilis* Fabricius 1792 |  |  |  | 22 | 1.80 | 0.0852 |  |  |  |
| *Carabus violaceus purpurascens* Fabricius 1787 | 11 | **2.73** | **0.0195** |  |  |  | 22 | **-2.45** | **0.0226** |
|  |  |  |  |  |  |  |  |  |  |
| **Rove beetles** |  |  |  |  |  |  |  |  |  |
| *Drusilla canaliculata* (Fabricius 1787) |  |  |  | 22 | -1.96 | 0.0625 |  |  |  |
| *Metopsia clypeata* (Müller 1821) |  |  |  | 22 | **2.60** | **0.0164** | 22 | **-2.10** | **0.0474** |
| *Ocypus olens* (O. Müller 1764) |  |  |  | 22 | 1.92 | 0.0675 | 22 | -2.02 | 0.0552 |
|  |  |  |  |  |  |  |  |  |  |
| **Orthoptera** |  |  |  |  |  |  |  |  |  |
| *Gryllus campestris* Linnaeus 1758 | 11 | **2.91** | **0.0142** |  |  |  |  |  |  |
|  |  |  |  |  |  |  |  |  |  |
| **Spiders** |  |  |  |  |  |  |  |  |  |
| *Alopecosa pulverulenta* (Clerck 1757) | 11 | **-2.48** | **0.0304** |  |  |  |  |  |  |
| *Argenna subnigra* (O.P.-Cambridge 1861) |  |  |  | 22 | **2.49** | **0.0207** | 22 | **-2.15** | **0.0428** |
| *Aulonia albimana* (Walckenaer 1805) | 11 | **-3.33** | **0.0067** |  |  |  | 22 | **-2.77** | **0.0112** |
| *Cnephalocotes obscurus* (Blackwall 1834) | 11 | **2.32** | **0.0408** | 22 | **2.52** | **0.0196** | 22 | **-2.52** | **0.0193** |
| *Drassodes pubescens* (Thorell 1856) | 11 | **-2.26** | **0.0451** |  |  |  |  |  |  |
| *Drassyllus pumilus* (C.L. Koch 1839) |  |  |  |  |  |  | 22 | **3.02** | **0.0063** |
| *Hahnia nava* (Blackwall 1841) | 11 | -1.83 | 0.0951 |  |  |  | 22 | -1.87 | 0.0751 |
| *Ozyptila claveata* (Walckenaer 1837) |  |  |  | 22 | 2.00 | 0.0576 |  |  |  |
| *Pardosa bifasciata* (C.L. Koch 1834) | 11 | -2.09 | 0.0608 |  |  |  |  |  |  |
| *Pardosa monticola* (Clerck 1757) | 11 | **2.28** | **0.0434** |  |  |  |  |  |  |
| *Pardosa pullata* (Clerck 1757) | 11 | **-2.73** | **0.0196** | 22 | 1.79 | 0.0865 |  |  |  |
| *Trochosa terricola* Thorell 1856 | 11 | -2.02 | 0.0682 |  |  |  |  |  |  |
|  |  |  |  |  |  |  |  |  |  |
| **Woodlice** |  |  |  |  |  |  |  |  |  |
| *Trachelipus rathkii* (Brandt 1833) | 11 | **3.20** | **0.0085** |  |  |  |  |  |  |
