# Supplementary material for: Diverse Effects of a Seven-Year Experimental Grassland Fragmentation on Major Invertebrate Groups
Source: PLoS One. 2016 Feb 18;11(2):e0149567. doi: 10.1371/journal.pone.0149567 (PMC4758731; doi:10.1371/journal.pone.0149567)
Supplement: S2 Table — (DOC) [file pone.0149567.s003.doc]

**Supporting information for Braschler & Baur “Diverse Effects of a Seven-Year Experimental Grassland Fragmentation on Major Invertebrate Groups”**

**S2 Table.** Summaries of the full models^a^ for species density and individual density of all groups combined and each focal group separately showing results for the fixed effects fragmentation treatment (fragments vs. control plots), plot size (large vs. small) and the interaction fragment treatment*plot size^b^ during early and late collection periods. The findings for the early period are generally similar to those for the full dataset reported in **Table 2**. However, results for the late period show more differences. These differences may partly be explained by different seasonal activity patterns resulting from the hotter and drier weather conditions and lower plant productivity during mid-to late summer in grasslands on shallow soils. However, depletion of individuals through repeated pitfall trapping for 14 weeks may also have played a role.

**A) Data from first three collecting events (6 May to 17 June)**

|  | Treatment | | | Plot size | | | Interaction | | |
| --- | --- | --- | --- | --- | --- | --- | --- | --- | --- |
|  | df | t | p | df | t | p | df | t | p |
| Species density |  |  |  |  |  |  |  |  |  |
| All Groups | 11 | -1.41 | 0.19 | 22 | 0.00 | > 0.99 | 22 | -0.06 | 0.95 |
| Ants | 11 | -1.15 | 0.27 | 22 | -0.43 | 0.67 | 22 | 0.03 | 0.98 |
| Orthopterans | 11 | 2.16 | 0.0534 | 22 | -0.66 | 0.52 | 22 | 0.73 | 0.47 |
| Ground beetles | 11 | -0.99 | 0.34 | 22 | 0.81 | 0.42 | 22 | -0.54 | 0.60 |
| Rove beetles | 11 | -0.86 | 0.41 | 22 | 0.11 | 0.28 | 22 | -1.45 | 0.16 |
| Gastropods | 11 | -0.39 | 0.70 | 22 | -0.67 | 0.51 | 22 | 1.73 | 0.10 |
| Spiders | 11 | **-2.70** | **0.0208** | 22 | 0.07 | 0.95 | 22 | -0.40 | 0.69 |
| Woodlice | 11 | **2.81** | **0.0169** | 22 | 0.99 | 0.33 | 22 | -1.43 | 0.17 |
|  |  |  |  |  |  |  |  |  |  |
| Individual density |  |  |  |  |  |  |  |  |  |
| All Groups | 11 | -1.16 | 0.27 | 22 | 1.23 | 0.23 | 22 | -1.24 | 0.23 |
| Ants | 11 | -0.69 | 0.50 | 22 | 1.25 | 0.23 | 22 | -0.24 | 0.81 |
| Orthopterans | 11 | **3.03** | **0.0115** | 22 | -0.26 | 0.80 | 22 | -0.55 | 0.59 |
| Ground beetles | 11 | -0.51 | 0.62 | 22 | 0.83 | 0.42 | 22 | -0.85 | 0.41 |
| Rove beetles | 11 | -0.64 | 0.53 | 22 | 0.98 | 0.34 | 22 | -0.83 | 0.42 |
| Gastropods | 11 | -0.49 | 0.63 | 22 | -1.02 | 0.32 | 22 | 1.03 | 0.31 |
| Spiders | 11 | **-3.03** | **0.0115** | 22 | 0.97 | 0.34 | 22 | -1.86 | 0.08 |
| Woodlice | 11 | **2.98** | **0.0125** | 22 | 0.81 | 0.42 | 22 | -1.16 | 0.26 |

**B) Data from last three collecting events (1 July to 12 August)**

|  | Treatment | | | Plot size | | | Interaction | | |
| --- | --- | --- | --- | --- | --- | --- | --- | --- | --- |
|  | df | t | p | df | t | p | df | t | p |
| Species density |  |  |  |  |  |  |  |  |  |
| All Groups | 11 | 0.04 | 0.97 | 22 | -1.09 | 0.29 | 22 | 1.64 | 0.12 |
| Ants | 11 | 0.02 | 0.99 | 22 | -0.40 | 0.70 | 22 | 0.04 | 0.97 |
| Orthopterans | 11 | 1.11 | 0.29 | 22 | 0.41 | 0.69 | 22 | 1.32 | 0.20 |
| Ground beetles | 11 | **3.56** | **0.0044** | 22 | 1.74 | 0.10 | 22 | -1.98 | 0.06 |
| Rove beetles | 11 | -0.50 | 0.63 | 22 | -1.28 | 0.21 | 22 | 1.13 | 0.27 |
| Gastropods | 11 | -1.14 | 0.28 | 22 | **-3.28** | **0.0034** | 22 | **3.95** | **0.0007** |
| Spiders | 11 | -0.43 | 0.68 | 22 | 1.35 | 0.19 | 22 | -0.86 | 0.40 |
| Woodlice | 11 | 1.86 | 0.09 | 22 | 0.87 | 0.39 | 22 | -0.59 | 0.56 |
|  |  |  |  |  |  |  |  |  |  |
| Individual density |  |  |  |  |  |  |  |  |  |
| All Groups | 11 | 0.41 | 0.69 | 22 | -0.70 | 0.49 | 22 | 0.73 | 0.47 |
| Ants | 11 | -1.05 | 0.32 | 22 | 0.38 | 0.71 | 22 | 0.49 | 0.63 |
| Orthopterans | 11 | 1.52 | 0.16 | 22 | 0.62 | 0.54 | 22 | 0.34 | 0.74 |
| Ground beetles | 11 | **3.14** | **0.0093** | 22 | 1.08 | 0.29 | 22 | -1.38 | 0.18 |
| Rove beetles | 11 | -0.98 | 0.35 | 22 | -1.91 | 0.07 | 22 | 1.45 | 0.16 |
| Gastropods | 11 | 1.05 | 0.32 | 22 | **-2.86** | **0.0090** | 22 | 1.65 | 0.11 |
| Spiders | 11 | -0.88 | 0.40 | 22 | 1.48 | 0.15 | 22 | -0.71 | 0.49 |
| Woodlice | 11 | 1.53 | 0.15 | 22 | 0.47 | 0.64 | 22 | 0.18 | 0.86 |

^a^ The full model also accounted for the nested structure of the design and included the random factors site and block. Dependent variables were log_10_-transformed or log_10_(x + 1)-transformed for analysis.

^b^ Significant effects are shown in bold font. Positive t-values for treatment indicate groups that reached higher densities in fragments than in control plots, while negative t-values indicate groups whose densities were lower in fragments. The significant interaction term for gastropod species density was caused by the smaller fragments having increased species density than the larger fragments, while no difference was found between control plots of different size.
