# Supplementary material for: Diverse Effects of a Seven-Year Experimental Grassland Fragmentation on Major Invertebrate Groups
Source: PLoS One. 2016 Feb 18;11(2):e0149567. doi: 10.1371/journal.pone.0149567 (PMC4758731; doi:10.1371/journal.pone.0149567)
Supplement: S1 Text — (DOCX) [file pone.0149567.s004.docx]

**Supporting information for Braschler & Baur “Diverse Effects of a Seven-Year Experimental Grassland Fragmentation on Major Invertebrate Groups”**

**S1 Text. Methods and sources for habitat preferences**

**Gastropods**

Data for humidity preferences of gastropods were derived from Kerney *et al*. [1983] and Falkner *et al*. [2001].

**Ants**

Habitat preferences were obtained from Seifert [2007]. Vegetation composition and density, temperature and humidity changed in fragments, especially in the edge zone of fragments [Dolt et al. 2005]. We therefore focused on indicator values associated with preferences for these environmental conditions (indicators for temperature (T-M), humidity (F-M), and plant density (PD-M) as given on pages 104–105 in Seifert [2007]). Information on ranges of these indicator values was not used, as they were not available for all species. A species not listed in the table was omitted from the analyses (*Myrmecina graminicola*).

**Ground beetles**

Data on humidity preferences were extracted from Luka *et al*. [2009]. Humidity preferences for our species fell into 4 categories: hygrophilous (adapted for wet environments), mesophilous (no clear preference), xerophilous (adapted to a very dry environment), and steno-xerophilous (adapted to a very dry environment with a narrow niche).

**Rove beetles**

Data on preferences for humidity were compiled by H. Luka and were based on his work in progress with P. Nagel for the “Coleoptera, Staphilinidae der Schweiz. Ecology – Atlas” [Luka & Nagel, in prep]. Humidity preferences were: hygrophilous, mesophilous, and xerophilous.

**References**

Dolt C., Goverde M. & Baur B. (2005) Effects of experimental small-scale habitat fragmentation on above-ground and below-ground plant biomass in calcareous grasslands. Acta Oecol. 27, 49–56.

Falkner G., Obrdlik P., Castella E. & Speight M. C. D. (2001) Shelled Gastropoda of Western Europe. Munich, Germany: Friedrich-Held_Gesellschaft.

Kerney M. P., Cameron R. A. D. & Jungbluth J. H. (1983) Die Landschnecken Nord- und Mitteleuopas. Hamburg, Germany: Paul Parey.

Luka H., Nagel P., Feldmann B., Luka A. & Gonseth Y. (2009) Checkliste der Kurzflügelkäfer der Schweiz (Coleoptera: Staphilinidae ohne Pselaphinae). Mitt. Schweiz. Entomol. Ges. 82, 61–100.

Seifert B. (2007) Die Ameisen Mittel- und Nordeuropas. Tauer, Germany: Lutra – Verlags- und Vertriebsgesellschaft.
