# Supplementary material for: Diverse Effects of a Seven-Year Experimental Grassland Fragmentation on Major Invertebrate Groups
Source: PLoS One. 2016 Feb 18;11(2):e0149567. doi: 10.1371/journal.pone.0149567 (PMC4758731; doi:10.1371/journal.pone.0149567)
Supplement: S2 Text — (DOCX) [file pone.0149567.s005.docx]

**Supporting information for Braschler & Baur “Diverse Effects of a Seven-Year Experimental Grassland Fragmentation on Major Invertebrate Groups”**

**S2 Text. Species list.** Study sites are: M Movelier, N Nenzlingen, V Vicques

**Gastropods**

*Aegopinella nitens* (Michaud 1831) M

*Arion distinctus* J. Mabille 1868 M, N, V

*Arion intermedius* Normand 1852 M, N

*Arion vulgaris* Moquin-Tandon 1855 M, N, V

*Boettgerilla pallens* Simroth 1912 N

*Carychium tridentatum* (Risso 1826) M

*Cochlicopa lubrica* (O.F. Muller 1774) M, N, V

*Cochlodina laminata* (Montagu 1803) N

*Cochlostoma septemspirale* (Razoumowsky 1789) M

*Deroceras reticulatum* (O.F. Muller 1774) M, N, V

*Euconulus fulvus* (O. F. Muller 1774) M, N

*Helicella itala* (Linnaeus 1758) M, N, V

*Helix pomatia* Linnaeus 1758 M, N

*Monachoides incarnatus* (O. F. Muller 1774) N

*Nesovitrea hammonis* (Ström 1765) M, N, V

*Punctum pygmaeum* (Draparnaud 1801) M, N, V

*Pupilla muscorum* (Linnaeus 1758) N, V

*Succinella oblonga* (Draparnaud 1801) M

*Trochulus sericeus* (Draparnaud 1801) M, N, V

*Vallonia costata* (O. F. Muller 1774) M, N

*Vallonia excentrica* Sterki 1893 M, N, V

*Vertigo pygmaea* (Draparnaud 1801) M, N, V

Vitrina pellucida (O. F. Muller 1774) M, N

**Ants**

*Camponotus ligniperda* (Latreille 1802) M

*Formica cunicularia* Latreille 1798 M, N, V

*Formica pratensis* Retzius 1783 M, N, V

*Formica rufa* Linnaeus 1761 N

*Formica rufibarbis* Fabricius 1793 M, N, V

*Formica sanguinea* Latreille 1798 M, V

*Lasius flavus* (Fabricius 1782) M, N, V

*Lasius fuliginosus* (Latreille 1798) M, N

*Lasius niger* (Linnaeus 1758) N

*Lasius paralienus* Seifert 1992 M, N, V

*Myrmecina graminicola* (Latreille 1802) N, V

*Myrmica rubra* (Linnaeus 1758) M

*Myrmica ruginodis* Nylander 1846 M, N

*Myrmica sabuleti* Meinert 1861 M, N, V

*Myrmica scabrinodis* Nylander 1846 M, N, V

*Myrmica schencki* Emery 1895 M, N, V

*Myrmica specioides* Bondroit 1918 N, V

*Polyergus rufescens* (Latreille 1798) N, V

*Ponera coarctata* (Latreille 1798) M, N

*Solenopsis fugax* (Latreille 1798) N, V

*Strumigenys baudueri* (Emery 1875) N

*Tapinoma erraticum* (Latreille 1798) M, N

*Tapinoma subboreale* Seifert 2012 V

*Tetramorium caespitum* (Linnaeus 1758) M, N, V

*Themnothorax* sp. N

**Ground beetles**

*Abax ovalis* (Duftschmid 1812) N, V

*Abax parallelepipedus* (Piller & Mitterpacher 1783) M, N, V

*Abax parallelus* (Duftschmid 1812) M

*Amara aenea* (De Geer 1774) N

*Amara convexior* Stephens 1828 M

*Amara lunicollis* Schiodte 1837 N

*Anchmenus dorsalis* (Pontoppidan 1763) N

*Anisodactylus nemovariagus* (Duftschmid 1812) N

*Badister meridionalis* Puel 1925 M, N, V

*Calathus fuscipes* (Goeze 1777) M, N, V

*Callistus lunatus* (Fabricius 1775) M, N, V

*Carabus auratus* Linnaeus 1761 V

*Carabus cancellatus* Illiger 1798 V

*Carabus convexus* Fabricius 1775 M

*Carabus coriaceus* Linnaeus 1758 N, V

*Carabus monilis* Fabricius 1792 M, N, V

*Carabus nemoralis* O. F. Müller 1764 M, V

*Carabus problematicus* Herbst 1786 M, V

*Carabus violaceus purpurascens* Fabricius 1787 N, V

*Cicindela campestris* Linnaeus 1758 M, N

*Diachromus germanus* (Linnaeus 1758) N

*Harpalus dimidiatus* (P. Rossi 1790) M, N, V

*Harpalus latus* (Linnaeus 1758) M

*Harpalus rubripes* (Duftschmid 1812) N, V

*Leistus piceus* Fröhlich 1799 V

*Licinus depressus* (Paykull 1790) V

*Molops piceus* (Panzer 1793) M, V

*Panagaeus bipustulatus* (Fabricius 1775) M

*Parophonus maculicornis* (Duftschmid 1812) N

*Poecilus cupreus* (Linnaeus 1758) M, N, V

*Pterostichus burmeisteri* Heer 1838 M

*Pterostichus madidus* (Fabricius 1775) M, V

*Pterostichus ovoideus* (Sturm 1824) M, N

*Semiophonus signaticornis* (Duftschmid 1812) N

**Rove beetles**

*Anotylus nitidulus* (Gravenhorst 1802) N

*Anotylus rugosus* (Fabricius 1775) M

*Anotylus tetracarinatus* (Block 1799) N, V

*Astenus procerus* (Gravenhorst 1806) N

*Astenus gracilis* (Paykull 1789) M

*Atheta* sp. M, N, V

*Callicerus rigidicornis* (Erichson 1839) N

*Dinaraea angustula* (Gyllenhall 1810) M, N, V

*Drusilla canaliculata* (Fabricius 1787) M, N, V

*Metopsia clypeata* (Müller 1821) M, N, V

*Mycetoporus clavicornis* (Stephens 1832) V

*Mycetoporus lepidus* Gravenhorst 1802 N

*Ocypus aenocephalus* (De Geer 1774) M

*Ocypus olens* (O. Müller 1764) M, N, V

*Ocypus ophthalmicus* (Scopoli 1763) V

*Ocypus picipennis* (Fabricius 1793) M, N, V

*Ontholestes murinus* (Linnaeus 1758) V

*Oxypoda brachyptera* (Stephens 1832) V

*Paederus littoralis* Gravenhorst 1802 M, N, V

*Philonthus lepidus* (Gravenhorst 1802) N, V

*Philonthus varians* (Paykull 1789) V

*Platydracus fulvipes* (Scopoli 1763) N

*Platydracus stercorarius* (Olivier 1795) N, V

*Quedius semiobscurus* (Marsham 1802) N

*Quedius* sp. N

*Rugilus erichsoni* (Fauvel 1867) N

*Sepedophilus immaculatus* (Stephens 1832) M

*Staphylinus dimidiaticornis* Gemminger 1851 M, N

*Stenus ochropus* Kiesenwetter 1858 M, N

*Sunius bicolor* (Olivier 1795) N

*Tachyporus chrysomelinus* (Linnaeus 1758) V

*Tachyporus dispar* (Paykull 1789) N

*Tachyporus pusillus* Gravenhorst 1806 N

*Tasgius melanarius* (Heer 1839) V

*Xantholinus elegans* (Olivier 1795) N

*Xantholinus linearis* (Olivier 1795) N

*Xantholinus longiventris* Heer 1839 N

*Zyras limbatus* (Paykull 1789) M, N, V

*Zyras similis* (Märkel 1845) M, N, V

**Orthopterans**

*Gryllus campestris* Linnaeus 1758 M, N, V

**Spiders**

*Agroeca cuprea* Menge 1873 M

*Alopecosa accentuata* (Latreille 1817) V

*Alopecosa cuneata* (Clerck 1757) M, N, V

*Alopecosa pulverulenta* (Clerck 1757) M, N, V

*Alopecosa striatipes* (C.L. Koch 1837) V

*Alopecosa trabalis* (Clerck 1757) M, V

*Apostenus fuscus* Westring 1851 N

*Arctosa lutetiana* (Simon 1876) M, N

*Argenna subnigra* (O.P.-Cambridge 1861) N, V

*Asagena phalerata* (Panzer 1801) N, V

*Atypus piceus* (Sulzer 1776) N, V

*Aulonia albimana* (Walckenaer 1805) M, N, V

*Bathyphantes gracilis* (Blackwall 1841) N

*Callilepis schuszteri* (Herman 1879) M

*Cheiracanthium virescens* (Sundevall 1833) V

*Cicurina cicur* (Fabricius 1793) N

*Clubiona neglecta* O.P.-Cambridge 1862 M, N

*Cnephalocotes obscurus* (Blackwall 1834) M, N, V

*Dicymbium nigrum brevisetosum* (Blackwall 1834) N

*Dipoena melanogaster* (C.L. Koch 1837) M

*Drassodes cupreus* (Blackwall 1834) M, V

*Drassodes lapidosus* (Walckenaer 1802) M

*Drassodes pubescens* (Thorell 1856) M, N, V

*Drassyllus praeficus* (L. Koch 1866) M, N, V

*Drassyllus pumilus* (C.L. Koch 1839) N, V

*Drassyllus pusillus* (C.L. Koch 1833) M, N, V

*Dysdera erythrina* (Walckenaer 1802) M, N, V

*Enoplognatha thoracia* (Hahn 1833) M, N

*Episinus truncatus* Latreille 1809 N

*Euophrys frontalis* (Walckenaer 1802) M, N, V

*Euryopis flavomaculata* (C.L. Koch 1836) M

*Evarcha arcuata* (Clerck 1757) N

*Evarcha falcata* (Clerck 1757) M

*Gongylidiellum latebricola* (O.P.-Cambridge 1871) N

*Hahnia nava* (Blackwall 1841) M, N, V

*Haplodrassus kulczynskii* (Lohmander 1942) M, V

*Haplodrassus signifer* (C.L. Koch 1839) M, N, V

*Harpactea hombergi* (Scopoli 1763) N

*Harpactea lepida* (C.L. Koch 1838) M, N, V

*Heliophanus cupreus* (Walckenaer 1802) M, N

*Heliophanus flavipes* (Hahn 1832) N, V

*Histopona torpida* (C.L. Koch 1837) M, N

*Hypsosinga sanguinea* (C.L. Koch 1844) N, V

*Lasaeola prona* (Menge 1868) V

*Eratigena picta* (Simon 1870) M

*Agyneta affinis* (Kulczynski 1898) N

*Agyneta mollis* (O.P.-Cambridge 1871) N

*Agyneta rurestris* (C.L. Koch 1836) N, V

*Mermessus trilobatus* (Emerton 1882) M, N, V

*Micaria formicaria* (Sundevall 1831) M, N, V

*Micaria fulgens* (Walckenaer 1802) N

*Micaria pulicaria* (Sundevall 1831) M, N

*Micrargus subaequalis* (Westring 1851) M, N, V

*Microneta viaria* (Blackwall 1841) M, N

*Myrmarachne formicaria* (De Geer 1778) M, N, V

*Nematogmus sanguinolentus* (Walckenaer 1842) N, V

*Oedothorax fuscus* (Blackwall 1834) M

*Ozyptila atomaria* (Panzer 1801) N, V

*Ozyptila claveata* (Walckenaer 1837) M, N

*Ozyptila pullata* (Thorell 1875) V

*Ozyptila scabricula* (Westring 1851) N, V

*Ozyptila simplex* (O.P.-Cambridge 1862) N

*Pachygnatha degeeri* Sundevall 1830 M, N, V

*Panamomops mengei* (Simon 1926) M, N

*Pardosa bifasciata* (C.L. Koch 1834) N, V

*Pardosa hortensis* (Thorell 1872) M, N, V

*Pardosa monticola* (Clerck 1757) N, V

*Pardosa palustris* (Linnaeus 1758) N, V

*Pardosa prativaga* L. Koch 1870 N

*Pardosa pullata* (Clerck 1757) M, N, V

*Pardosa saltans* (Topfer-Hofmann 2000) M, N, V

*Phlegra fasciata* (Hahn 1826) N

*Phrurolithus festivus* (C.L. Koch 1835) M, N

*Phrurolithus minimus* C.L. Koch 1839 N

*Phrurolithus nigrinus* (Simon 1878) V

*Pisaura mirabilis* (Clerck 1757) N, V

*Pocadicnemis juncea* Locket & Millidge 1953 M, N

*Pocadicnemis pumila* (Blackwall 1841) M

*Robertus lividus* (Blackwall 1836) M

*Robertus neglectus* (O.P.-Cambridge 1871) V

*Scotina palliardii* (L. Koch 1881) V

*Sibianor aurocinctus* (Ohlert 1865) M, V

*Stemonyphantes lineatus* (Linnaeus 1758) N, V

*Talavera aequipes* (O.P.-Cambridge 1871) N, V

*Talavera inopinata* Wunderlich 1993 M, N, V

*Tenuiphantes tenuis* (Blackwall 1852) M, N, V

*Trochosa robusta* Simon 1876 N, V

*Trochosa ruricola* De Geer 1778 N

*Trochosa terricola* Thorell 1856 M, N, V

*Walckenaeria antica* (Wider 1834) M, N, V

*Walckenaeria obtusa* Blackwall 1836 N

*Xysticus cristatus* (Clerck 1757) V

*Xysticus erraticus* (Blackwall 1834) N, V

*Xysticus kochi* Thorell 1872 M, N, V

*Xysticus robustus* (Hahn 1832) V

*Zelotes apricorum* (L. Koch 1876) M

*Zelotes exiguus* (Muller & Schenkel 1895) N, V

*Zelotes latreillei* (Simon 1878) M, N

*Zelotes petrensis* (C.L. Koch 1839) M, V

*Zodarion italicum* (Canestrini 1868) N

*Zora nemoralis* (Blackwall 1861) M, N, V

*Zora silvestris* Kulczynski 1897 N

*Zora spinimana* (Sundevall 1833) N

**Woodlice**

*Armadillidium opacum* (C.L. Koch 1841) M

*Ligidium hypnorum* (Cuvier 1792) M, N, V

*Oniscus asellus* Linnaeus 1758 N, V

*Philoscia muscorum* (Scopoli 1763) N

*Trachelipus rathkii* (Brandt 1833) M, N, V
